# Damage Model of Steel Fiber-Reinforced Coal Gangue Concrete under Freeze–Thaw Cycles Based on Weibull Distribution

**DOI:** 10.3390/ma16206775

**Published:** 2023-10-19

**Authors:** Yaohui Cheng, Li Sun, Yongjing Li, Mengxin Liu, Ruixia He, Xiaoying Jin, Huijun Jin

**Affiliations:** 1 School of Civil Engineering and Transportation, Permafrost Institute, China-Russia Joint Laboratory of Cold Regions Engineering and Environment, Northeast Forestry University, Harbin 150040, China; cyhddfc@126.com (Y.C.); liumengxin@nefu.edu.cn (M.L.); hjjin@nefu.edu.cn (H.J.); 2 Liaoning Provincial Key Laboratory of Coal Gangue Resource Utilization and Energy-Saving Building Materials, School of Civil Engineering, Liaoning Technical University, Fuxin 123000, China; lyjsdyt@126.com; 3 Department of Housing and Urban-Rural Development of Heilongjiang Province, Harbin 150070, China; 4 State Key Laboratory of Frozen Soils Engineering, State Key Laboratory of Cryosphere Science, Northwest Institute of Eco-Environment and Resources, Chinese Academy of Sciences, Lanzhou 730000, China; heruixia1026@163.com

**Keywords:** steel fiber-reinforced coal gangue concrete (SCGC), freeze–thaw cycle (FTC), flexural properties, microscopic analysis, damage evolution model

## Abstract

**Highlights:**

**What are the main findings?**

- Mechanical properties of steel fiber-reinforced coal gangue concrete (SCGC) are tested, proving the effectiveness of strength enhancement of SCGC and helping to expand the application range of SCGC.
- The damage evolution model of SCGC under freeze–thaw cycles (FTCs) is established by introducing damage variables of coal gangue and SCGC.

**What is the implication of the main finding?**

- This study provides key experimental data and a theoretical basis for a wider range of proper uti-lization of coal gangue in cold regions.

**Abstract:**

In order to improve the utilization rate of coal gangue and expand the application range of coal gangue concrete (CGC), a certain proportion of steel fiber was added to the concrete, and the freeze–thaw cycles (FTCs) and flexural tests were used to explore the effects of different mass replacement rates of coal gangue (0%, 25%, 50%, 75%, and 100%) and different proportions of the volumetric blending of the steel fiber (0%, 0.8%, 1.0%, and 1.2%) on the frost resistance of steel fiber-reinforced CGC (SCGC). The governing laws of mass loss rate, relative dynamic elastic modulus and load–midspan deflection curve were obtained on the base of the analysis of testing results. The damage mechanisms of the SCGC under the FTCs were analyzed using the results of scanning electron microscopy (SEM). Based on the Lemaitre’s strain equivalence principle and Krajcinovic’s vector damage theory, a damage evolution model of the SCGC under the FTCs was established by introducing the damage variable of the SCGC satisfying Weibull distribution. The results show an increasing mass loss rate of the SCGC and a decreasing relative dynamic elastic modulus with an increasing mass replacement rate of coal gangue. The proper content of the steel fiber can reduce the mass loss rate of concrete by 10~40% and the relative loss rate of dynamic elastic modulus of concrete by 2~8%, thus significantly improving the ductility and toughness of the concrete. The established damage evolution model is well validated by the experimental results, which further help to improve the modelling accuracy. This study provides key experimental data and a theoretical basis for a wider range of proper utilization of coal gangue in cold regions.

## 1. Introduction

Coal gangue is an associated product of coal. For every ten tons of coal yield, one ton of coal gangue is produced. As a major and largest coal producer in the world, China had an annual total output of 4.56 billion tons of coal in 2022, accounting for 51.8% of the total output of coal in the world, with an increase of 10.5% in comparison with that in 2021 [1,2,3]. So far, the accumulation of coal gangue in China has reached as high as 5.5~5.6 billion tons, forming many gangue hills with a total areal extent of up to 160 km^2^, seriously affecting land use and reclamation and environmental management. Therefore, it is urgent to improve the utilization rate of coal gangue [4,5,6,7,8,9,10]. Using coal gangue, instead of natural gravel, to prepare concrete not only reduces the pollution of coal gangue in the soil, water, and atmospheric environments but also saves natural nonrenewable materials.

At present, many studies have been conducted on coal gangue concrete (CGC). It is found that, due to the need for secondary crushing of coal gangue for coarse aggregates, many internal microcracks are unavoidable. Compared with natural gravel, coal gangue has some particular properties, such as low bearing strength, high brittleness, and rough surfaces. Replacing natural gravel with coal gangue to prepare concrete will reduce the working performance, strength, and durability of concrete [11,12]. Therefore, an innovative method has been proposed for improving the performance of CGC, boosting the utilization rate of coal gangue and augmenting the application range of CGC. Similar to the methods for improving the performance of natural gravel concrete, the proposed improvement method can be mainly divided into two aspects [13]. On the one hand, it is to raise the content of cementitious material in concrete, thereby increasing the bonding between the cementitious material and the coarse aggregate. For example, Haruna et al. (2022) mixed a certain amount of nano-silica into the cement to make steel fiber-reinforced concrete [14]. Their study found that the proper incorporation of nano-silica could improve the impact resistance performance of steel fiber-reinforced concrete (SFC), and the impact damage factor of SFC satisfies the Weibull distribution. Jin et al. (2022) explored the influences of different coal gangues and metakaolin on the mechanical properties of concrete. Their results showed a declined compressive strength of concrete with the incorporation of coal gangue and a boosted compressive strength of the concrete with metakaolin [15]. On the other hand, a certain volume of fiber is added to the concrete to mitigate the crack propagation in the concrete [16,17,18,19]. In order to explore the influence of fiber on the lifespan of concrete, de Alencar Monteiro et al. (2022) prepared an SFC for testing its mechanical properties. Their results showed a positive effect of the steel fiber addition on the lifespan of the concrete, and the Weibull distribution was used to establish the lifespan prediction model of the SFC [20]. Xia et al. (2022) conducted axial compression tests on short columns of concrete strengthened with coal gangue and steel fiber, which was confined in a glass fiber-reinforced polymer (GFRP) tube, and found a more obvious improvement effect of corrugated fibers on the ultimate axial strain of short columns in comparison with that of hooked fibers [21].

It was found that, in the service process of concrete, compared with the loading test, the durability test was a greater challenge. In China, coal mines are mainly distributed in the northern regions, including Northeast, Northwest, and North China. From the perspective of local materials, one of the major concerns of the application of CGC in the northern regions is whether the frost resistance durability meets the application requirements [22,23,24,25]. Therefore, in order to expand the application range of CGC in northern China, an appropriate proportion of fiber can be incorporated. Qiu et al. (2020) explored the effect of coal gangue on the frost resistance durability of CGC [26]. The results showed that in order to ensure that CGC meets the requirements of frost resistance durability, the replacement rate of coal gangue should be less than 40%. The established model for damage evolution of CGC under freeze–thaw cycles (FTCs) can truly and effectively reflect the development processes of freeze–thaw damage to CGC. Guan et al. (2022) explored the influences of bending load and the FTC conditions on mechanical properties of CGC and found that the larger the bending stress ratio of CGC, the more rapid the compressive strength and relative dynamic elastic modulus decline [27].

The existing research has mainly focused on the frost resistance durability and damage evolution of CGC, and most of the results are consistent with each other or among themselves. However, there are few studies on the influences of fiber incorporation on the frost resistance durability of CGC, mechanistical analysis, and damage constitutive model. This situation has greatly limited the practical application of steel fiber-reinforced coal gangue concrete (SCGC). Therefore, on the basis of existing studies, in this paper, we explored the influences of coal gangue mass substitution rate and steel fiber volumetric fraction on the frost resistance of the SCGC. The influence mechanisms of various factors on the mechanical properties of concrete are analyzed from the microscopic view using a scanning electron microscope (SEM). Based on the damage theory and Krajcinovic’s vector damage theory, the damage variable of the SCGC satisfying the Weibull distribution is introduced to derive the damage constitutive model of the SCGC. This study can help to provide a theoretical basis for promoting the resource utilization of coal gangue and expanding the scope of use of CGC.

## 2. Raw Materials and Test Methods

### 2.1. Raw Materials

**Figure 1:**
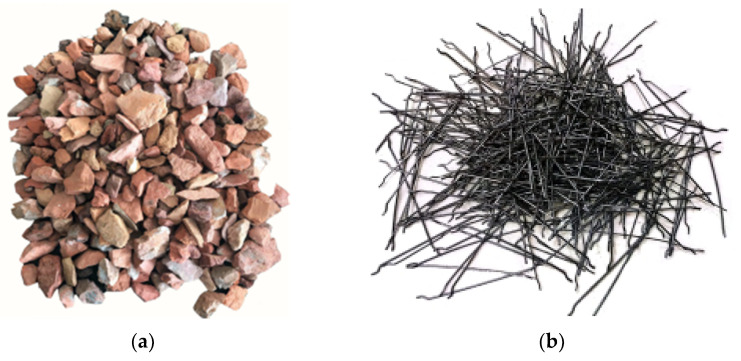
Coal gangue and steel fiber used in the test. Notes: (**a**) Coal gangue after spontaneous combustion, and (**b**) end hook-type steel fiber.

Spontaneous combustion coal gangue from Xinqiu, Fuxin in western Liaoning Province, Northeast China, and granite gravel from Zhangwu, Fuxin in western Liaoning Province, Northeast China, with particle sizes ranging from 5 to 20 mm, were chosen as coarse aggregates. Their major physical properties are shown in Table 1. End hook-type steel fiber, with a length of 37.5 mm and a diameter of 0.75 mm, was selected *per se* (Figure 1). Natural fluvial sand was chosen as a fine aggregate, with its parameters listed in Table 2. The Fuxin Daying brand P·O 42.5 of ordinary Portland cement (Portland, OR, USA) was selected, with the initial and final setting time of 80 and 240 min, respectively. Its physical and mechanical properties are shown in Table 3. The polycarboxylate superplasticizer was selected as a water reducer.

### 2.2. Mix Ratio of SCGC

In China, the national codes JGJ55-2019 [28] and JG/T-2015 [29] and other relevant codes were consulted and complied for the mix ratio of SCGC used in the FTC test and bending resistance test. The literature [30] review indicates that when the volumetric fraction of the steel fiber incorporated in the cement mortar was less than 0.50%, it had a minor enhancement effect on the mechanical properties of the concrete; when the content was greater than 2.0%, the steel fibers in the mortar could not be evenly distributed, resulting in its agglomeration remained in the mortar and inhibiting the enhancement of the concrete. Thus, in this test, the range of the volumetric fraction of the steel fiber was determined by a multi-party inspection. The specific mix ratios are shown in Table 4.

### 2.3. Specimen Preparation

**Figure 2:**
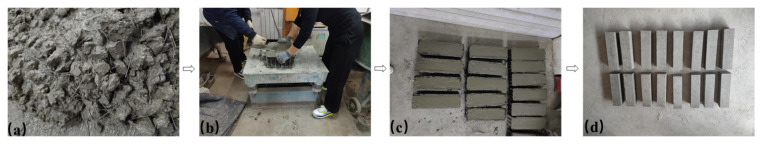
The processes of preparing test specimens. Notes: (**a**) Mixing, (**b**) pouring and vibratory molding, (**c**) curing, and (**d**) demolding.

According to the *National Standard of China for Test Methods of Physical and Mechanical Properties of Concrete* (GB/T 50081-2019) [31], the SCGC specimens were made with the mix ratios shown in Table 4. The flexural test specimens were 100 × 100 × 400 mm in size, and three groups of parallel specimens were prepared under each test condition. Six cube specimens of 100 × 100 × 100 mm in size were made for each mix ratio to test the cube compressive strength and splitting tensile strength of the SCGC. The results of the cube compressive strength and splitting tensile strength testing of the specimens are shown in Table 5. The test process is shown in Figure 2.

### 2.4. Test Methods

#### 2.4.1. FTC Test

**Figure 3:**
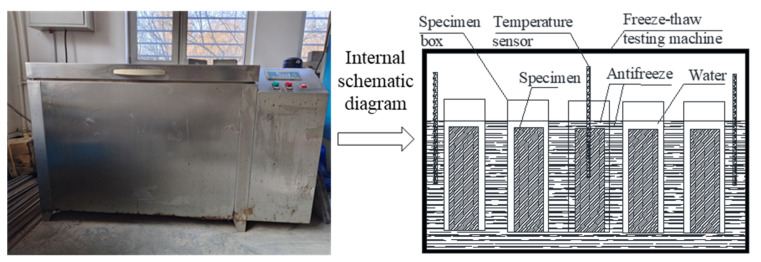
Freeze–thaw cycle (FTC) test chamber (**left**) and its configurations (**right**) of this study.

Referring to the specifications of the *National Standard of China for Test Methods of Long-Term Performance and Durability of Ordinary Concrete* (GB/T 50082-2009) [32], the molded SCGC specimens were cured in the standard curing room for 24 d, then they were taken out and put in water at a temperature range of 18~22 °C for 4 d, and the FTC test was subsequently carried out with a quick-freezing method. The minimum and maximum temperatures in the test box were strictly maintained between −20 and −16 °C and between 3 and 7 °C, respectively. The time of one FTC was set at 8 h. The corresponding test instrument is shown in Figure 3. Each time, after 25 FTCs, the transverse fundamental frequency *f*_n_ and mass *W*_n_ were measured. When at least one of the following conditions occurred, the test was terminated: (1) 300 FTCs; (2) reduction in the relative dynamic elastic modulus to 60% of the original value, and; (3) a 5% mass loss rate.

#### 2.4.2. Bending Resistance Test

Every time, after 25 FTCs, a group of concrete with each ratio needed to be taken for flexural test. The test was carried out according to the flexural strength test method of concrete as specified in the GB/T 50081-2019 [31]. The loading rate was set at 0.05 MPa/s, and the load and midspan deflection during the failure process of the specimen were recorded according to the following principles:

(1) The arithmetic average of the test results of the three parallel specimens was regarded as the deflective strength of this group of the specimens.
(2) If the difference between either the minimum or maximum and mean values of the test results of three parallel specimens was greater than 15% of the mean value, the two values (minimum and maximum) should be discarded, and the mean value should be regarded as the test value for this group of the concrete specimens. If the difference between the maximum and mean values and that between the minimum and mean values were all greater than 15% of the mean value, the test results were considered invalid, and the group test had to be repeated.

#### 2.4.3. Scanning Electron Microscope (SEM) Test

After the bending resistance test, the SCGC was crushed to prepare concrete samples smaller than 1 cm^3^ in volume, in order to ensure the smoothness of the observed position. During the test, the specimens were completely dried and strictly sealed for gold spray observation. The SEM instrument used during the experiment was the Czech TESCAN MIRA LMS produced by Tesken Co., Ltd. in Brno, Czech Republic.

#### 2.4.4. Evaluation Index Calculation Method

(1) The test piece mass loss rate is calculated as follows:

(1) $$M=\frac{m_{0}-m_{n}}{m_{0}}\times 100\%$$

where *M* is the mass loss rate, in %, to an accuracy of ±0.01; *m*_0_ is the mass of the specimen before the FTCs, in kg; and *m_n_* is the mass of the specimen after *n* FTCs, in kg.

**Figure 4:**
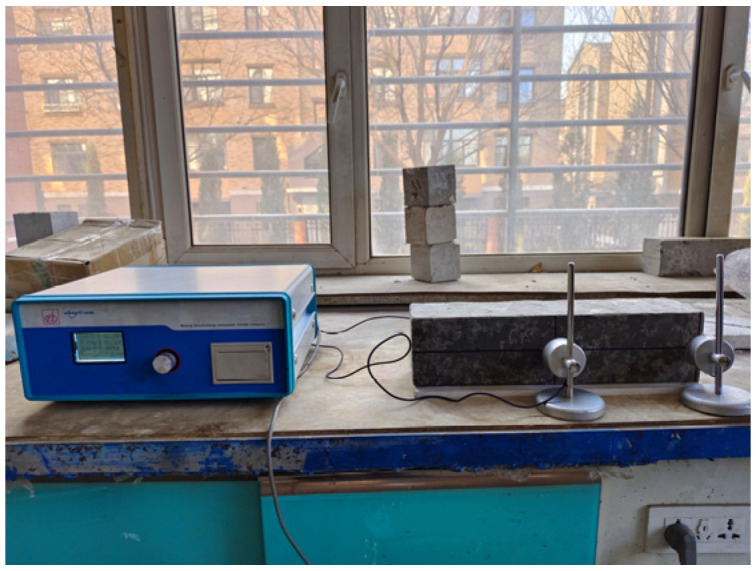
DT-W18 dynamic elastic modulus tester and test process of this study.

(2) The relative dynamic modulus of elasticity of the specimen is calculated as follows:

(2) $$E_{A}=\frac{f_{0}^{2}}{f_{n}^{2}}\times 100\%$$

where *E*_A_ is the relative dynamic elastic modulus, in %, to an accuracy of ±0.1, and *f*_0_ and *f_n_* are the transverse fundamental frequencies of the specimen before and after *n* FTCs, in Hz. The test instrument is a DT-W18 dynamic elastic modulus tester produced by Beijing Digital Yilong Instrument Co., Ltd. in Beijing China (Figure 4).

## 3. Results and Analysis

### 3.1. Frost Resistance of SCGC

**Figure 5:**
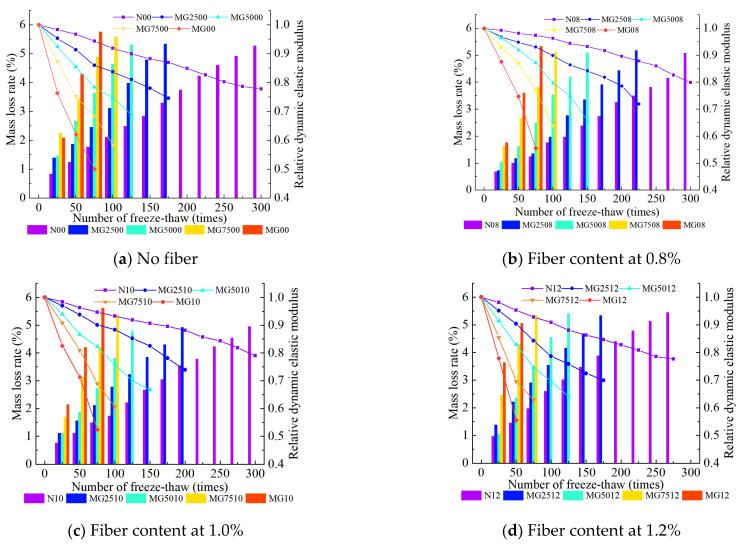
Mass loss and relative dynamic elastic modulus of steel fiber-reinforced coal gangue concrete (SCGC) with fiber content at 0% (**a**), 0.8% (**b**), 1.0% (**c**), and 1.2% (**d**).

Figure 5 shows the influences of the coal gangue mass substitution rate on the mass loss rate and relative dynamic elastic modulus of the SCGC. With the increasing number of FTCs, the mass loss rates of the natural gravel concrete, CGC, and SCGC increase gradually; however, the relative dynamic elastic modulus decreases gradually, and the change trend is slow first and then rapid, indicating increased frost cracking of the natural gravel concrete, CGC, and SCGC. Upon 25 FTCs, the frost resistance index of each group of concrete changes only slightly; when the number of FTCs reaches 50 times, the minimum mass loss rate in the natural gravel concrete is N08, which is 1.01%, and its relative dynamic elastic modulus decreases to 0.98. The maximum mass loss rate in the natural gravel concrete is N12, which is 1.41%, and its relative dynamic elastic modulus decreases to 0.95. The mass loss rate of concrete with a 25% coal gangue mass substitution rate is in the range of 1.18% (MG2508)~2.21%(MG2512), while the relative dynamic elastic modulus is in the range of 0.90 (MG2512)~0.94 (MG2508). The mass loss rate of concrete with a coal gangue mass substitution rate of 100% is in the range of 3.61% (MG08)~5.06% (MG12), while the relative dynamic elastic modulus is in the range of 0.55 (MG12)~0.74 (MG08), when the freeze–thaw damage occurs. Evidently, the proportional increase in the coal gangue for replacing the natural gravel can reduce the frost resistance of the concrete. This is because there are many pores in the spontaneous combustion coal gangue, which has undergone secondary crushing before the test, resulting in more initial cracks in its interior than in the natural gravel. Therefore, in the water-saturated state, compared with natural gravel concrete, more water can be stored. The larger the mass replacement rate of the coal gangue, the more water stored, and the greater the volume change after the specimen freezing. The more cracks that lead to further expansion and development of the SCGC, the better the macroscopic performance as revealed by a larger mass loss rate of the SCGC and a smaller relative dynamic elastic modulus with the increasing coal gangue mass replacement rate with the same number of the FTCs. Therefore, with the same number of FTCs, the frost resistance index of the concrete with a coal gangue mass substitution rate of 25% is closer to that of the natural gravel concrete. Therefore, to have the highest frost resistance of the SCGC, the coal gangue mass substitution rate should be set to 25%.

At the same mass substitution rate of the coal gangue, with an increasing volumetric fraction of the steel fiber, the mass loss rate and relative dynamic elastic modulus of the SCGC corresponding to the same number of the FTCs decrease first and then increase, and the larger the mass substitution rate of the coal gangue, the more obvious the phenomenon. When the mass replacement rate of the coal gangue is set at 25%, taking 100 FTCs as an example, and when the volumetric fraction of the steel fiber is set at 0% (MG2500), 0.8% (MG2508), 1.0% (MG2510), and 1.2% (MG2512), the mass loss rate is 3.12%, 1.98% (reduced by 36.54%), 2.77% (reduced by 11.22%), and 3.54% (increased by 13.46%) respectively; the relative dynamic elastic modulus is 0.83, 0.90 (increased by 8.43%), 0.88 (increased by 6.02%), and 0.78 (reduced by 6.02%). When the mass replacement rate of the coal gangue reaches 50%, the SCGC with 0.8% and 1.0% of the steel fiber can still withstand 125 FTCs, and the frost resistance index of the SCGC with a 0.8% steel fiber volumetric fraction increases the greatest. The reason for this phenomenon is that the addition of the appropriate amount of steel fiber (0.8%) can enhance the connections in the SCGC structure due to the bridging effect, which slows the development of initial cracks in the SCGC, thus fundamentally reducing the internal damage of the material. However, the excessive addition of steel fiber (e.g., 1.0% or 1.2%) will lead to its accumulation and agglomeration in the concrete structure, resulting in that the cement mortar cannot be closely connected with the internal structure of the concrete and thus in an increase in the initial microcracks of the CGC. Thus, the damage is aggravated, leading to the conditions favorable for the emergence and development of cracks. Therefore, if the service life of the SCGC in cold regions is expected to be longer, the volumetric fraction of the steel fiber should be set to 0.8%.

In summary, in order to produce SCGC with the best frost resistance, the optimal coal gangue mass replacement rate should be 25%, while the optimal steel fiber volumetric fraction should be 0.8%.

### 3.2. Load–Midspan Deflection Curves

**Figure 6:**
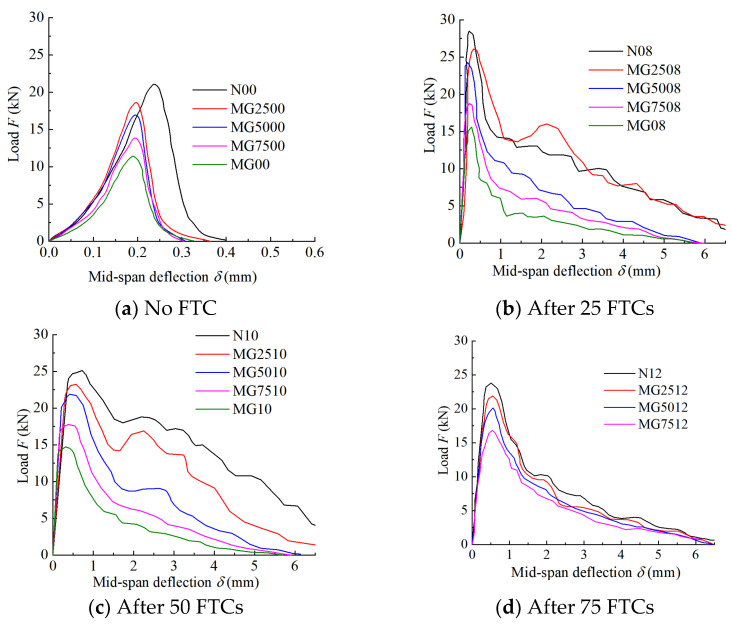
Load–deflection curve of steel fiber-reinforced coal gangue concrete (SCGC) with 0%, 25%, 50%, 75%, and 100% coal gangue after the number of freeze–thaw cycles of 0 (**a**), 25 (**b**), 50 (**c**), and 75 (**d**).

When the coal gangue completely replaced the natural gravel, the SCGC with 1.2% steel fiber failed after 75 freeze–thaw cycles. Therefore, in order to ensure the accuracy of the test results, only those for 0, 25, 50 and 75 FTCs were analyzed. The specific results are shown in Figure 6.

It can be seen from Figure 6 that the load–deflection curves of the natural gravel concrete, CGC, and SCGC differ remarkably. Figure 6a is the case for the natural gravel concrete and CGC with 0 FTCs. During the loading process, the curves showed a rapid upward trend. After the peak load, it decreased immediately and sharply, and the descending section was steeper than the rising section, indicating an abrupt and brittle failure. The minimal (0.2–0.3 mm) midspan deflection at the peak load indicates a low toughness of the natural gravel concrete and coal gangue concrete, but the toughness of the natural gravel concrete is better than that of the coal gangue concrete. In Figure 6b–d, with an increasing coal gangue content in the SCGC and with the same number of FTCs and steel fiber content, the bearing capacity, ductility, and toughness of the concrete show a downward trend. After the peaked deflection–load curve, the rate of decline is positively correlated with the content of the coal gangue. Compared with natural gravel, coal gangue is more brittle and has some original damage due to secondary crushing. Therefore, its own bearing capacity is lower than that of natural gravel, and its strength decreases faster than that of natural gravel. The aggregate is more easily broken, and the water absorption rate of coal gangue is higher than that of natural gravel. Therefore, the frost heave characteristics of coal gangue are more obvious under the FTCs. The macroscopic performance is that the test curve shows a decreasing trend with the increasing mass substitution rate of the coal gangue. Therefore, the bearing capacity, ductility, and toughness of the concrete with a coal gangue mass replacement rate of 25% are greater than those with other replacement rates.

When the volumetric fraction of the steel fiber was set at 0.8%, 1.0%, or 1.2%, the load–deflection curves of the concrete specimens showed a rapid upward trend before the peak load, but the midspan deflection of the peak load position of the CGC fell within 0.2~0.3 mm, while that of the SCGC increased to 0.5~1 mm. Evidently, the concrete specimen with a steel fiber volumetric fraction of 0.8% had the most obvious improvement. After the peak load, the load–midspan deflection curve of the SCGC was smoother than that of the natural gravel concrete or CGC, and the curve showed a fluctuating downward trend. When the mass replacement rate of the coal gangue was no higher than 50%, and the number of FTCs was no more than 75 times, the load–midspan deflection curve continued to have the second, lower, peak after the first, main, peak, and after the second peak, it showed a fluctuating downward trend. However, compared to the natural gravel concrete and CGC, the SCGC had an obvious improvement in ductility and toughness: the maximum midspan deflection of the natural gravel concrete and CGC was 0.3 and 0.4 mm, respectively. After the incorporation of the steel fiber, the maximum midspan deflection of the SCGC became greater than 5 mm. The ductility and toughness of the concrete were improved the most at a volumetric fraction of the steel fiber at 0.8%. The reason for such an improvement was that during the bending process of the SCGC, the steel fiber possibly played a key role in connecting the concrete on both sides of the crack and hindered the continuous development of the crack upon the failure of the concrete specimen, when the energy required for destroying the SCGC was greater, thereby improving its toughness. When the volumetric fraction of the steel fiber was excessively large, the steel fiber would be unevenly distributed in the concrete mortar. A part of the steel fiber agglomerated, instead of being tightly wrapped, in the cement mortar. Macroscopically, the ductility and toughness of the concrete declined due to the presence of an excessively high volumetric fraction of the steel fiber.

In summary, in order to improve the ductility and toughness of SCGC, the coal gangue mass substitution rate should be set to 25%, and the volumetric fraction of the steel fiber to 0.8%. These conclusions are consistent with the analysis results of the SCGC frost resistance described in Section 3.1.

### 3.3. Mechanistical Analysis at a Microscopic Scale

(a) Microstructure of concrete after curing

**Figure 7:**
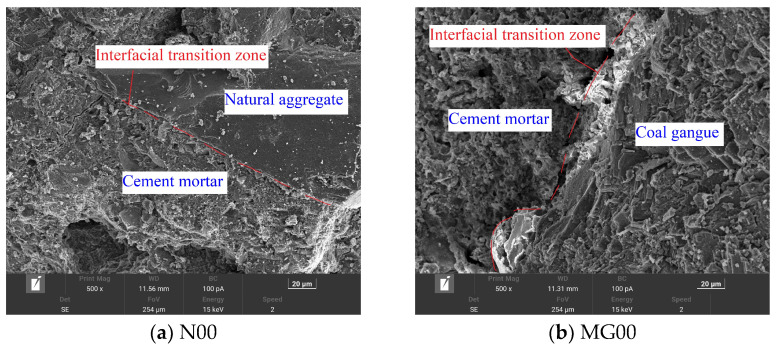
Scanning electron microscopic (SEM) images of concrete without freeze–thaw cycle. Notes: (**a**) N00 (natural crushed stone concrete without steel fiber) and (**b**) MG00 (coal gangue concrete (CGC) without steel fiber).

The SEM was used for observing and analyzing the microstructure for the specimens of the natural gravel concrete and the CGC with the optimal mix ratio after curing (Figure 7).

It can be clearly seen from Figure 7 that compared with the CGC, the interface transition zone between the natural gravel concrete and the cement mortar is closely bonded, with a very stable structure and few pores at a relatively dense interface, indicating a tightly wrapped coarse aggregate by the cement mortar and increased bonding. Microscopically, this may well explain why the natural gravel concrete has higher strength, stronger bending resistance, and better durability than CGC. The interface transition zone between the coarse aggregate of the CGC and the cement mortar is more obvious, and there are some small cracks. The surface of the aggregate is rough, and the content of needle-like minerals is more than that of natural gravel, indicating poor bonding between coal gangue and the cement mortar, resulting in a lower interface strength between the coarse aggregate and the cement mortar in comparison with that between the natural gravel and the cement mortar. Due to the relatively loose structure and larger water absorption of the coal gangue, the cracks in the interface transition zone will preserve more water. Therefore, compared with the natural gravel as a coarse aggregate, the coal gangue will absorb more water in the concrete structure. Therefore, compared with the natural gravel concrete, the CGC has lower strength, lower frost resistance, and lower ductility.

(b) Microstructure of concrete after FTCs

**Figure 8:**
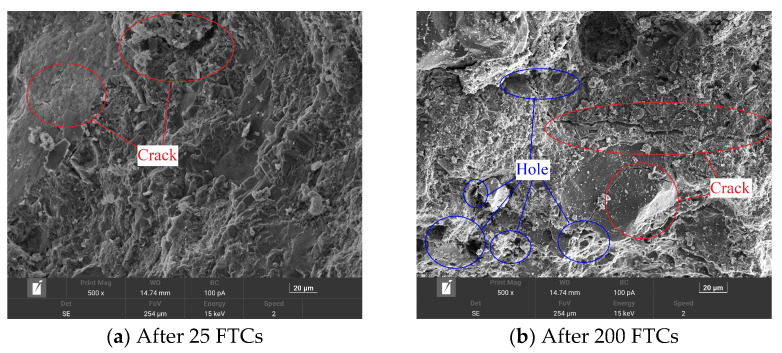
Scanning electron microscope images of natural gravel concrete after 25 (**a**) and 200 (**b**) freeze–thaw cycles (FTCs).

**Figure 9:**
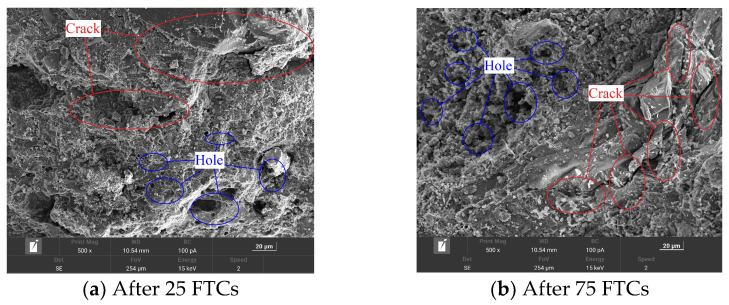
Scanning electron microscope images of coal gangue concrete after 25 (**a**) and 75 (**b**) freeze–thaw cycles.

**Figure 10:**
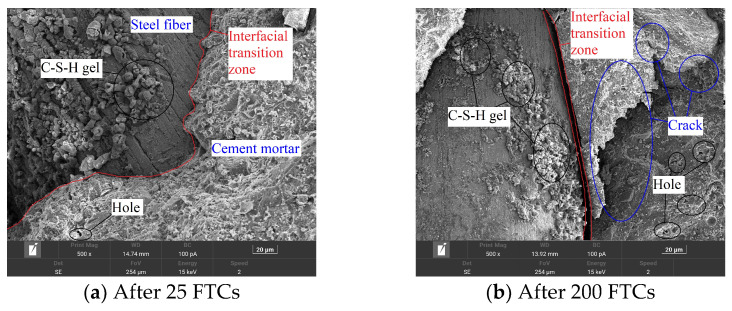
Scanning electron microscope images of steel fiber-reinforced coal gangue concrete after two different numbers of freeze–thaw cycles (FTCs). Notes: FTCs of (**a**) 25 and (**b**) 200 times.

SEM results are often used to explain the change process in the natural gravel concrete, CGC, and SCGC with the number of FTCs from the microscopic point of view. In this study, the influences of the FTCs, coal gangue aggregate, and steel fiber incorporation on concrete are qualitatively analyzed, as shown in Figure 8, Figure 9 and Figure 10.

It can be seen from Figure 8 that the micromorphology of natural gravel concrete differs substantially after 25 and 200 FTCs. When the natural gravel concrete undergoes fewer FTCs, its structure is relatively compact, with only a few microcracks. The crack width is about 1 μm in diameter, the structure is relatively dense and compact, with a small number of tiny holes in the cement slurry. When the number of FTCs reaches 200 times, evidently wide and long cracks appear. The width of the cracks increases to 3~4 μm; the structure become looser; the holes area increases visibly, and; small holes start to occur everywhere. The hole diameter is in the range of 5~10 μm. Evidently, FTCs can reduce the compactness inside the SCGC, resulting in a series of cracks. Macroscopically, after many FTCs, the tested specimen is damaged to a greater extent.

From Figure 9a, after 25 FTCs, large cracks start to appear in the CGC. The width of the cracks reaches about 2 μm, with some large holes. The maximum hole diameter reaches 5 μm, with a loose structure. When the number of FTCs reaches 75 times (Figure 9b), cracks appear directly on the surface of the coarse aggregate and run through the coarse aggregate. The crack width increases to 4–5 μm. The whole structure becomes looser, with ubiquitous holes and cracks. Therefore, because the coal gangue coarse aggregate has the characteristics of low strength and more microdamage, the microdamage can store more water. After its use as a coarse aggregate to configure the concrete, the damage of the FTCs to the coal gangue concrete destroys not only the bonding structure between the cement mortar and the coal gangue coarse aggregate but also the coal gangue coarse aggregate itself. This can also be used to explain the poor frost resistance and low strength of the CGC in the macrotest.

It can be seen from Figure 10 that with a smaller number of FTCs, the cement mortar and the steel fiber are closely connected, with a good interface transition zone but without obvious cracks. The surface of the steel fiber attaches to many flocculent hydration products (e.g., C-S-H gel). Some small holes appear in the cement matrix, without obvious changes in the interfacial transition zone between the steel fiber and cement. After the number of FTCs increases, visible cracks, up to 62.3 μm, appear in the interface transition zone between the steel fiber and the cement matrix. The cement matrix also has a wide crack, expanding into the concrete, as compared with Figure 10a. The holes in Figure 10b are larger and denser, with conspicuous characteristics of inter-structural deterioration. It can be seen that FTCs can lead to microdamage inside the SCGC that continues to expand, weakening the SCGC. When the number of FTCs is small, the steel fiber and the concrete structure are closely connected. At this time, when the SCGC is subjected to a bending test, the steel fiber and the concrete structure jointly bear the load. When the load is small, there is an interaction force between the steel fiber and the concrete structure. When the load increases, some cracks start to appear in the more fragile cement mortar until the destruction of the concrete structure. After many FTCs, before the loading, due to the frost heaving effect of hole water freezing, cracks appear between the steel fiber and the cement matrix, resulting in declined bonding between the two. This, in turn, increases the brittleness of the SCGC and weakens the material. This observation agrees well with the results obtained from the macroscopic test in this study.

## 4. SCGC Frost Damage Modeling Based on Weibull Distribution

### 4.1. Modeling

There were two kinds of coarse aggregates (natural gravel and coal gangue) used in the SCGC. The coal gangue aggregate is characterized by low strength, some internal micro-damage, and a complex internal structure of the SCGC. Therefore, in order to more easily simulate the damage evolution of MG2508 (steel fiber-reinforced coal gangue concrete with a coal gangue mass substitution rate of 25% and a steel fiber volumetric fraction of 0.8%) under the FTCs, the following assumptions were made to build the damage model:

(1) Continual damage of the SCGC under bending;
(2) All coarse aggregates are evenly distributed in the SCGC;
(3) During bending, the SCGC structure is connected by interface microelements, which constitute the corresponding interface transition zone, and there is congenital damage.

According to the Lemaitre’s strain equivalence principle [33]:

(3) $$\sigma =E\varepsilon \left(1-D\right)$$

where *σ* is the effective stress; *ε* is the effective strain; *E* is the elastic modulus, and; *D*, the damage variable.

Under the FTCs, damage variable *D* of the SCGC includes two parts. The first part is the internal damage of the SCGC after the FTCs. Previous studies have found that the dynamic elastic modulus can more accurately describe the internal concrete damage after the FTCs [34,35]. Therefore, in this paper, the dynamic elastic modulus was selected as the damage evaluation index, and the degree of freeze–thaw damage *D*_f_ was determined according to the damage mechanics as follows:

(4) $$D_{f}=\frac{E_{d0}-E_{dn}}{E_{d0}}$$

where *E*_d0_ is the dynamic elastic modulus of the SCGC without an FTC, and; *E*_d*n*_ is the dynamic elastic modulus of the SCGC after *n* FTCs.

The second part is damage *D*_p_ of the SCGC under load. In the statistical damage model, the SCGC is usually considered as a series of defective microunits to satisfy the mandates of the Weibull distribution. Therefore, under the loading conditions, damage *D*_p_ of the material can be expressed as the ratio of the number of damaged microunits (*N_f_*) to the number of microunits of the material (*N*) [36]:

(5) $$D_{p}=\frac{N_{f}}{N}$$

When the load on the SCGC increases to its yield strength, the microelement damage inside the concrete reaches *N*_f_:

(6) $$N_{f}=\int_{0}^{\varepsilon }Nf\left(x\right)dx$$

Then, damage *D*_p_ can be further expressed as follows:

(7) $$D_{p}=\int_{0}^{\varepsilon }f\left(x\right)dx$$

The microscopic heterogeneity of deformation characteristics and mechanical properties of brittle materials, such as concrete and rock, obeys the law of the Weibull distribution [37]. That is, *f*(*x*) in Equation (7) is the probability density function corresponding to the Weibull distribution function satisfied by the damage caused by the load in the SCGC [38]:

(8) $$f\left(\varepsilon \right)=\frac{\beta }{\alpha }{\left(\frac{\varepsilon }{\alpha }\right)}^{\beta -1}\exp\left[-{\left(\frac{\varepsilon }{\alpha }\right)}^{\beta }\right]$$

where *α* and *β* are the scale and shape parameters, and *ε* is the effective strain.

Equation (9) can be obtained by substituting Equation (8) into Equation (7):

(9) $$D_{p}=1-\exp[-{\left(\frac{\varepsilon }{\alpha }\right)}^{\beta }]$$

By combining Equations (5) and (9), the formula of the damage variable of the SCGC under the FTCs is obtained:

(10) $$D=1-\left(1-D_{f}\right)\left(1-D_{p}\right)=1-\frac{E_{dn}}{E_{d0}}\exp[-{\left(\frac{\varepsilon }{\alpha }\right)}^{\beta }]$$

By bringing Equation (10) into Equation (3), the damage constitutive model of the SCGC before peak load under the FTCs can be obtained as follows

(11) $$\sigma =E\varepsilon \frac{E_{dn}}{E_{d0}}\exp[-{\left(\frac{\varepsilon }{\alpha }\right)}^{\beta }]$$

The stress–strain curve of the SCGC can be divided into the stages of non-destructive and damaged evolution. The former can be fitted by Equation (11). In order to establish the model of the damage evolution stage, it is assumed that when the concrete is subjected to deformation, the deformation of the fiber and the concrete is the same, without relative sliding or dislocation. Based on the Krajcinovic’s vector damage theory and the Clausius–Duhem inequality [37], the damage evolution equation of the SCGC is obtained:

(12) $$\sigma_{ij}=E\frac{E_{dn}}{E_{d0}}k\left[\begin{array}{l} 1+(C_{1}+C_{2})D\;\bar{v}+\frac{1}{2}C_{1}D\;\bar{v}+\frac{1}{2}C_{1}D\\ \bar{v}+\frac{1}{2}C_{1}D\;\;\;\;\;\;\;\;\;\;\;\;\;\;\;\;\;\;1\;\;\;\;\;\;\;\;\;\;\;\;\;\;\;\;\;\;\bar{v}\\ \bar{v}+\frac{1}{2}C_{1}D\;\;\;\;\;\;\;\;\;\;\;\;\;\;\;\;\;\;\bar{v}\;\;\;\;\;\;\;\;\;\;\;\;\;\;\;\;\;\;1 \end{array}\right]\varepsilon_{ij}$$

where *σ_ij_* is the stress tensor $\bar{v}=\frac{v}{1-v}$, $k=\frac{1-v}{\left(1+v\right)\left(1-2v\right)}$, and *ν* is the Poisson ratio; and *C*_1_ and *C*_2_ are the material constants. For the SCGC, *ν* = 0.2, *C*_1_ = 3.5, and *C*_2_ = 0.4; *ε_ij_* is the strain tensor.

According to the hypothesis, the growth of microcracks mainly occurs at the midpoint of the specimen in the vertical direction. According to the incremental formula, the following conclusions can be drawn:

(13) $$\left\{\begin{array}{l} d\sigma_{11}\\ 0\\ 0 \end{array}\right\}=E\frac{E_{dn}}{E_{d0}}k\left[\begin{array}{l} 1+(C_{1}+C_{2})D\;\bar{v}+\frac{1}{2}C_{1}D\;\bar{v}+\frac{1}{2}C_{1}D\\ \bar{v}+\frac{1}{2}C_{1}D\;\;\;\;\;\;\;\;\;\;\;\;\;\;\;\;\;\;1\;\;\;\;\;\;\;\;\;\;\;\;\;\;\;\;\;\;\bar{v}\;\\ \bar{v}+\frac{1}{2}C_{1}D\;\;\;\;\;\;\;\;\;\;\;\;\;\;\;\;\;\;\bar{v}\;\;\;\;\;\;\;\;\;\;\;\;\;\;\;\;\;\;1 \end{array}\right]\left\{\begin{array}{l} d\varepsilon_{11}\\ d\varepsilon_{22}\\ d\varepsilon_{33} \end{array}\right\}+\left[\begin{array}{l} C_{2}\varepsilon_{11}+\frac{1}{2}C_{1}(\varepsilon_{22}+\varepsilon_{33})\\ \frac{1}{2}C_{1}\varepsilon_{11}\\ \frac{1}{2}C_{1}\varepsilon_{11} \end{array}\right]dD$$

That is,

(14) $$d\varepsilon_{22}=d\varepsilon_{33}=-(1-v)\left[(\frac{v}{1-v}+\frac{1}{2}C_{1}D\;)d\varepsilon_{11}+\frac{1}{2}C_{1}\varepsilon_{11}dD\right]$$

where *σ*_11_ is the normal stress in the x direction; *ε*_11_, *ε*_22_, and *ε*_33_ are positive strains in x, y, and z directions, respectively.

Based on the Mohr–Coulomb criterion under bending conditions, a hyperbolic method is used to simplify the damage surface. At this time, the strain generated is the same as the damage evolution value.

(15) $$f\left(\varepsilon_{11},D\right)=d\varepsilon_{11}-dD=0$$

By combining Equations (14) and (15), the definite integral is obtained:

(16) $$\varepsilon_{22}=\varepsilon_{33}=-(1-v)\left[(\frac{v}{1-v}+\frac{1}{2}C_{1}D\;)\varepsilon_{11}+\frac{1}{2}C_{1}\varepsilon_{11}^{2}\right]$$

By substituting Equation (14) into Equation (16) and Equation (10) into Equation (13), we yield the following:

(17) $$d\sigma_{11}=E\frac{E_{dn}}{E_{d0}}k\left\{\begin{array}{l} \left[1+(C_{1}+C_{2})D+(\frac{v}{1-v}{)}^{2}+\frac{C_{1}Dv}{1-v}+{\left(\frac{1}{2}C_{1}D\right)}^{2}\right]d\varepsilon_{11}\\ +\left[C_{2}-v-\frac{C_{1}D}{2}+\frac{C_{1}vD}{2}+\frac{C_{1}Dv}{2\left(1-v\right)}+\frac{1}{4}C_{1}^{2}D\right]\varepsilon_{11}d\varepsilon_{11}\\ -\frac{1-v}{2}C_{1}\varepsilon_{{}_{11}}^{2}d\varepsilon_{11} \end{array}\right\}$$

Setting:

(18) $$\begin{array}{l} m=1+(C_{1}+C_{2})D+(\frac{v}{1-v}{)}^{2}+\frac{C_{1}Dv}{1-v}+{\left(\frac{1}{2}C_{1}D\right)}^{2}\\ n=C_{2}-v-\frac{C_{1}D}{2}+\frac{C_{1}Dv}{2}+\frac{C_{1}Dv}{2\left(1-v\right)}+\frac{1}{4}C_{1}^{2}D\\ q=-\frac{1-v}{2}C_{1} \end{array}$$

Then, Equation (17) can be modified into the following:

(19) $$d\sigma_{11}=E\frac{E_{dn}}{E_{d0}}k\left(md\varepsilon_{11}+n\varepsilon_{11}d\varepsilon_{11}-q\varepsilon_{11}^{2}d\varepsilon_{11}\right)$$

The definite integral of Equation (19) can be solved as follows:

(20) $$\sigma_{11}=E\frac{E_{dn}}{E_{d0}}k\left(md\varepsilon_{11}+\frac{n}{2}\varepsilon_{11}^{2}-\frac{q}{3}\varepsilon_{11}^{3}\right)$$

During the loading process, the SCGC is only subjected to bending load, so the constitutive damage equation of the SCGC under the FTCs is written as follows:

(21) $$\sigma =\left\{\begin{array}{l} E\varepsilon \frac{E_{dn}}{E_{d0}}\exp[-{\left(\varepsilon /\alpha \right)}^{\beta }]\\ E\frac{E_{dn}}{E_{d0}}k\left(md\varepsilon +\frac{n}{2}\varepsilon_{}^{2}-\frac{q}{3}\varepsilon_{}^{3}\right) \end{array}\right.\begin{array}{cc} \begin{array}{l} 0<\varepsilon <\varepsilon_{\mathrm{pk}}\\ \varepsilon_{\mathrm{pk}}<\varepsilon \end{array} & \end{array}$$

where *ε*_pk_ is the strain corresponding to the peak stress.

### 4.2. Distribution Parameterization and Model Validation

**Figure 11:**
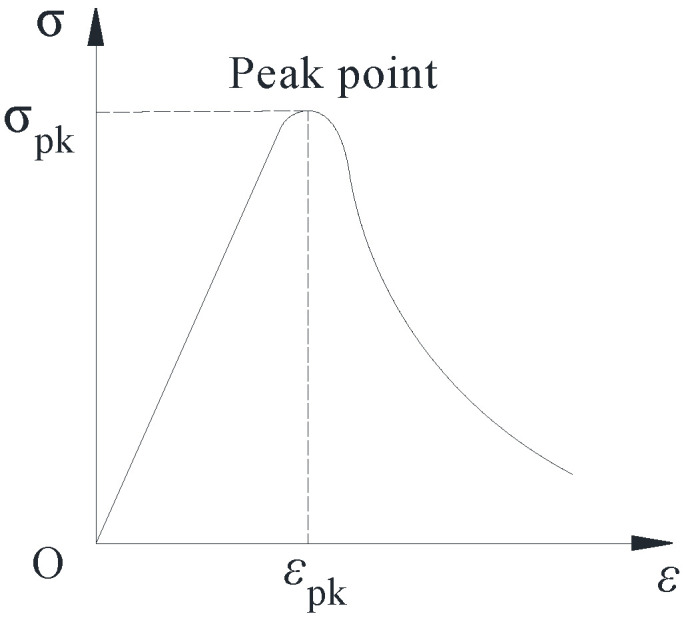
Schematic diagram of the stress–strain curve.

According to Equation (21) of the damage constitutive model of the SCGC, the dynamic elastic modulus *E*_d0_ of the specimen and the dynamic elastic modulus (*E*_d*n*_) of the SCGC after *n* FTCs were obtained from the test results. However, if we need to obtain the stress–strain curve of the SCGC under the FTCs, only the values of *α* and *β* are needed. According to Figure 11, there are the following geometric relationships on the stress–strain curve:

1. When *ε* = *ε*_pk_, *σ* = *σ*_pk_;
2. When *ε* = *ε*_pk_, *dσ*/*dε* = 0.

From relationship (1) in combination with Equation (11), it follows that

(22) $$\sigma_{\mathrm{pk}}=E\varepsilon_{\mathrm{pk}}\frac{E_{dn}}{E_{d0}}\exp[-{\left(\varepsilon_{\mathrm{pk}}/\alpha \right)}^{\beta }]$$

where *σ*_pk_ is the peak stress, and *ε*_pk_ is the peak strain.

From relationship (2) combined with Equation (22), it can be seen that

(23) $$\alpha =\varepsilon_{\mathrm{pk}}\cdot {\left(\frac{1}{\beta }\right)}^{-\frac{1}{\beta }}$$

(24) $$\beta =\frac{1}{\ln\left(\frac{E\varepsilon_{\mathrm{pk}}\frac{E_{\mathrm{dn}}}{E_{d0}}}{\sigma_{\mathrm{pk}}}\right)}$$

where *σ*_pk_ is the peak stress; *E* is the elastic modulus; *ε*_pk_ is the peak strain; *D* is the damage variable; *E*_d0_ is the dynamic elastic modulus of the SCGC without a FTC; *E*_d*n*_ is the dynamic elastic modulus of the SCGC after *n* FTCs; and *α* and *β* are the scale and shape factors.

During the loading process, the SCGC is in a state of compression on the one side and tension on the other, and the maximum stress point is at a certain position in the upper loading area of the specimen. The flexural failure at this position conforms to the constitutive relationship of concrete damage in the tension-bending state. The load–deflection curve of the relationship between load and displacement in the vertical direction of the SCGC can be transformed into the corresponding stress–strain curve through the corresponding relationship [39,40]. According to the principles of material mechanics:

(25) $$\varepsilon =\frac{6\delta h}{l^{2}}$$

(26) $$\sigma =\frac{My}{I}$$

(27) $$I=\frac{bh^{3}}{12}$$

(28) $$M=\frac{Pl}{4}$$

where *δ* is the midspan deflection, in mm; *l* is the distance between the left and right supports under the specimen, in mm; *h* is the height of the cross-section of the specimen, in this paper *h* = 100 mm; *M* is the bending moment of the specimen, in kN·m; *I* is the moment of inertia of the specimen, in m^4^; *b* is the width of the cross-section of the specimen, in mm; and *y* is the distance from the center of the moment of inertia to the neutral axis of the specimen, in mm, in this paper for *h*/2.

The corresponding stress–strain curve can be obtained by the load–midspan deflection curve obtained from the test through Equations (25)–(28), and the peak data can be obtained thereupon. Substituting them into Equations (23) and (24), the distributive parameters of each specimen can be obtained, as shown in Table 6.

**Figure 12:**
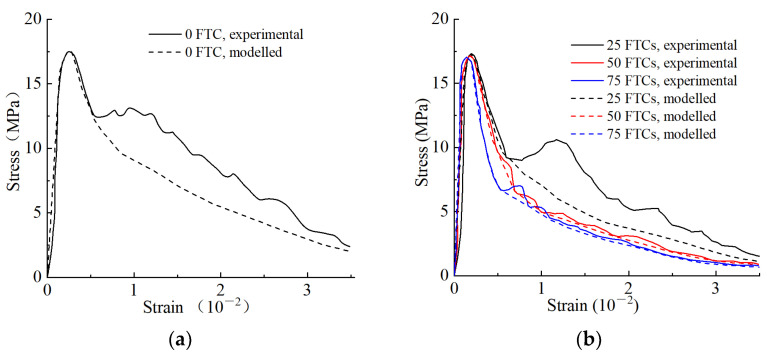

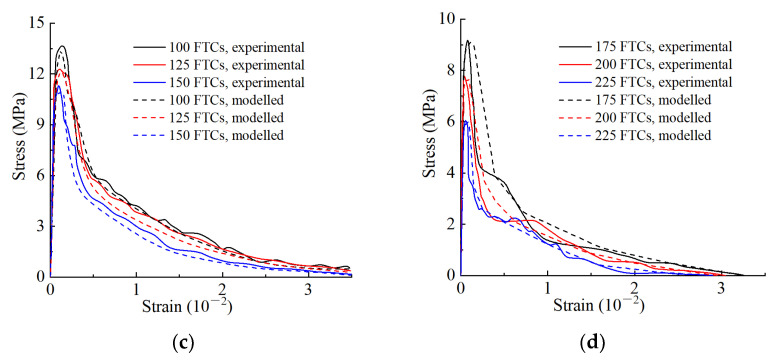
The comparison between the experimental and theoretical stress–strain curves of MG2508 (steel fiber-reinforced coal gangue concrete (SCGC) with a coal gangue mass substitution rate of 25% and a steel fiber volumetric content of 0.8%) with a different number of freeze–thaw cycles (FTCs). Notes: With the number of FTCs of 0 (**a**); 25, 50, and 75 (**b**); 100, 125, and 150 (**c**); and 175, 200, and 225 (**d**). The R^2^ (determination coefficient) values of FTCs at 0, 25, 50, and 75 were 0.924, 0.931, 0.947, and 0.953, respectively; those of FTCs at 100, 125, and 150 were 0.989, 0.952, and 0.964, respectively; and those of FTCs at 175, 200, and 225 were 0.946, 0.965, and 0.971, respectively.

Substituting the data from Table 6 into Equation (21), the simulated stress–strain curve of the SCGC can be obtained, and the stress–strain curve derived from the test results is plotted in the same diagram to further explore the accuracy of the damage constitutive model of the SCGC, as shown in Figure 12.

From the results shown in Figure 12, the determination coefficient (R^2^) of each model curve was greater than 0.9, indicating that the stress–strain curve of the SCGC specimen under the FTCs is in good agreement with that of the theoretical damage constitutive model, but there is a slight difference at the peak. This is mainly due to the differences in the individual samples, that is, the differences between the test and the simulated values resulting from the difference in the fiber content of the fracture surface. The measured flexural strength of the test is greater than the theoretically (model) calculated flexural strength, but the curve of the constitutive damage model before the peak is well fitted with the test data curve. Thus, the proposed constitutive damage model of SCGC under FTCs can more accurately describe the stress–strain changes in SCGC under FTCs. Therefore, the practicability and rationality of the model is validated herewith.

## 5. Conclusions

1. With an increasing mass substitution rate of coal gangue, the frost resistance durability of SCGC decreases. The mass loss and relative dynamic elastic modulus of SCGC can be significantly reduced. When the number of FTCs reaches 50 times, the mass loss rate of SCGC is in the range of 1.18% (MG2508)~5.06% (MG12), while the relative dynamic elastic modulus is in the range of 0.55 (MG12)~0.94 (MG2508). In order to make SCGC more frost-resistant, the mass substitution rate of coal gangue should be set to 25%.
2. With different volumetric fractions of steel fiber, the frost resistance index of SCGC varies. When the volumetric fraction of the steel fiber is 0.8% or 1.0%, the frost resistance index of SCGC increases obviously (the mass loss rate of concrete is reduced by 10~40%, and the relative dynamic elastic modulus is reduced by 2~8%). When the volumetric fraction of the steel fiber is 1.2%, the frost resistance of SCGC decreases (the mass loss rate of concrete increases by 16.2~74.6%, and the relative dynamic elastic modulus increases by 0.3~12.9%), but the incorporation of steel fiber can increase the ductility and toughness of SCGC (the maximum midspan deflection of the natural gravel concrete and CGC was 0.3 and 0.4 mm, respectively; after the incorporation of the steel fiber, the maximum midspan deflection of the SCGC became greater than 5 mm). In order to produce SCGC for cold regions with higher ductility and toughness, the volumetric fraction of the steel fiber should be set to 0.8%.
3. FTCs can increase the internal cracks and pore size of concrete, the interfacial transition zone between the coarse aggregate and the cement mortar, and the damage degree of coal gangue and destroy the connection between the steel fiber and the concrete structure, loosening the SCGC and macroscopically lowering the frost resistance index.
4. The stress–strain curves of the SCGC specimens under the FTCs agree well with those predicted by the theoretical constitutive damage model. This proves that the model can accurately describe the stress–strain changes in SCGC under FTCs.

## Figures and Tables

**Table 1:** Major physical properties of coarse aggregates in this study.

Category	Bulk Density (kg/m^3^)	Apparent Density (kg/m^3^)	Water Absorption (%)	Needle Flake Content (%)	Crushing Index (%)
Coal gangue	1073	2510	10.25	12.74	17.64
Natural gravel	1403	2840	0.92	5.83	5.81

**Table 2:** Fluvial sand parameters.

Detection Index	Fineness Modulus	Porosity (%)	Apparent Density (kg/m^3^)	Water Absorption (%)	Bulk Density (kg/m^3^)
Numerical value	2.71	43.12	2660	3.39	1420

**Table 3:** Physical and mechanical properties of cement.

Index	Specific Surface Area (m^2^/kg)	Loss on Ignition (%)	Compressive Strength (MPa)		Flexural Strength (MPa)	
			3 d	28 d	3 d	28 d
Numerical value	342	2.81	27.8	52.1	6.3	9.4

**Table 4:** Concrete ratio design.

Specimen Number	Strength Grade	Coal Gangue (kg)	Gravel Mass (kg)	Steel Fiber (kg)	Water (kg)	Cement (kg)	Sand (kg)	Water Reducer (kg)
N00	C30	0	902	0	195	390	800	7.8
N08		0	902	62				
N10		0	902	78				
N12		0	902	94				
MG2500		226	676	0				
MG2508		226	676	62				
MG2510		226	676	78				
MG2512		226	676	94				
MG5000		451	451	0				
MG5008		451	451	62				
MG5010		451	451	78				
MG5012		451	451	94				
MG7500		676	226	0				
MG7508		676	226	62				
MG7510		676	226	78				
MG7512		676	226	94				
MG00		902	0	0				
MG08		902	0	62				
MG10		902	0	78				
MG12		902	0	94				

Notes: The letter N denotes concrete with natural gravel as the coarse aggregate, and the letter combination MG denotes concrete with coal gangue as the coarse aggregate. MG25, MG50, MG75, and MG represent the specimens with coal gangue quality replacement rates of 25%, 50%, 75%, and 100%, respectively. In addition, in the name tags, the numbers 00, 08, 10, and 12 represent concrete with volumetric fractions of steel fiber at 0.0%, 0.8%, 1.0% and 1.2%, respectively.

**Table 5:** The SCGC specimens’ 28-day compressive strength and splitting strength.

Specimen Number	Compressive Strength (MPa)	Splitting and Tensile Strength (MPa)	Specimen Number	Compressive Strength (MPa)	Splitting and Tensile Strength (MPa)
N00	40.3	3.01	MG7500	32.3	2.61
N08	43.7	3.41	MG7508	35.1	2.96
N10	42.9	3.19	MG7510	32.8	2.74
N12	39.8	2.96	MG7512	31.9	2.59
MG2500	38.3	2.97	MG00	29.7	2.49
MG2508	42.3	3.33	MG08	30.8	2.61
MG2510	40.5	3.07	MG10	30.1	2.53
MG2512	37.8	2.95	MG12	29.5	2.44
MG5000	36.3	2.94			
MG5008	38.6	3.14			
MG5010	36.5	2.93			
MG5012	35.9	2.88			

**Table 6:** Mechanical properties and model parameters of the MG2508 specimen (steel fiber-reinforced coal gangue concrete (SCGC) with a coal gangue mass substitution rate of 25% and a steel fiber volumetric fraction of 0.8%).

Parameters	MG2508	Parameters	MG2508	Parameters	MG2508	Parameters	MG2508	Parameters	MG2508
*α*_0_	0.00143	*α*_50_	0.00139	*α*_100_	0.00133	*α*_150_	0.00119	*α*_200_	0.00108
*β*_0_	0.96	*β*_50_	0.93	*β*_100_	0.89	*β*_150_	0.8	*β*_200_	0.72
*α*_25_	0.00141	*α*_75_	0.00137	*α*_125_	0.00126	*α*_175_	0.00114	*α*_225_	0.00105
*β*_25_	0.95	*β*_75_	0.92	*β*_125_	0.84	*β*_175_	0.76	*β*_225_	0.7

Notes: The subscripts of *α* and *β* are the number of freeze–thaw cycles.

## Data Availability

Data will be made available on request.
